# Antimicrobial Dosing Recommendations in Pediatric Continuous Renal Replacement Therapy: A Critical Appraisal of Current Evidence

**DOI:** 10.3389/fped.2022.889958

**Published:** 2022-05-12

**Authors:** Gideon Stitt, Samuel Dubinsky, Andrea Edginton, Yuan-Shung V. Huang, Athena F. Zuppa, Kevin Watt, Kevin Downes

**Affiliations:** ^1^Center for Clinical Pharmacology, Children’s Hospital of Philadelphia, Philadelphia, PA, United States; ^2^School of Pharmacy, University of Waterloo, Waterloo, ON, Canada; ^3^Department of Biomedical and Health Informatics, Children’s Hospital of Philadelphia, Philadelphia, PA, United States; ^4^Department of Anesthesiology and Critical Care Medicine, Perelman School of Medicine, University of Pennsylvania, Philadelphia, PA, United States; ^5^Division of Critical Care Medicine, Children’s Hospital of Philadelphia, Philadelphia, PA, United States; ^6^Division of Clinical Pharmacology, Department of Pediatrics, University of Utah, Salt Lake City, UT, United States; ^7^Division of Infectious Diseases, Children’s Hospital of Philadelphia, Philadelphia, PA, United States; ^8^Department of Pediatrics, Perelman School of Medicine, University of Pennsylvania, Philadelphia, PA, United States

**Keywords:** continuous renal replacement therapy (CRRT), pediatrics, critical care, pharmacokinetics, pediatric intensive care unit

## Abstract

**Objectives:**

Continuous renal replacement therapy (CRRT) is commonly employed in critically ill children and is known to affect antimicrobial pharmacokinetics. There is a lack of readily available, evidence-based antimicrobial dosing recommendations in pediatric CRRT. This study aims to quantify commonly used antimicrobial drugs in pediatric CRRT and identify gaps between contemporary literature-based dosing recommendations and those presented in a frequently used dosing reference.

**Methods:**

The Pediatric Health Information System (PHIS) database was queried from July 1, 2018 through June 30, 2021 to identify admissions in which antimicrobials were billed on the same day as CRRT. Drugs of interest were selected if at least 10% of admission involved administration on at least one CRRT day, with additional clinically important antimicrobials selected by the authors. A comprehensive literature search was performed to identify antimicrobial pharmacokinetic (PK) studies in children for each selected drug. For identified articles, dosing recommendations were extracted and compared to those in a popular tertiary dosing reference (Lexi-Comp Online database). The level of agreement of the dosing recommendations was assessed.

**Results:**

77 unique antimicrobial agents were identified amongst 812 admissions from 20 different PHIS hospitals. Fifteen antimicrobials were billed on the same day as CRRT in ≥10% of admissions, with 4 additional drugs deemed clinically relevant by the authors. Twenty PK studies were identified for these 19 drugs, and dosing recommendations were included in 8 (42.1%) of them. Seventeen agents (89.5%) had some type of CRRT-specific dosing guidance in Lexi-Comp, with only 1 directly based on a pediatric CRRT study. For the 8 agents with PK data available, Lexi-Comp recommendations matched primary literature dosing guidance in 3 (37.5%). Two (25%) lacked agreement between the Lexi-Comp and primary literature, and the remaining 3 (37.5%) had partial agreement with multiple dosing regimens suggested in the primary literature and at least one of these regimens recommended by Lexi-Comp.

**Conclusion:**

Significant gaps exist in the data supporting antimicrobial dosing recommendations for children receiving CRRT. Future studies should focus on antimicrobial dosing in pediatric CRRT, emphasizing provision of robust data from which dosing recommendations can be promptly incorporated into tertiary dosing references.

## Introduction

Pediatric sepsis is a significant cause of morbidity and mortality, occurring at an estimated rate of 1.2 million cases per year (1). Associated mortality rates range from 4 to 50% depending on severity of illness, risk factors, and geographical location (2). Specifically, need for dialysis is an independent predictor of mortality in pediatric patients with sepsis (3). Broad-spectrum antibiotics are commonly administered to children with sepsis, especially during the empiric treatment phase, however, pediatric dosing recommendations during dialysis are limited for many antibiotics (4–6).

In addition to a lack of data supporting specific dosing during pediatric continuous renal replacement therapy (CRRT) for many commonly used antimicrobial agents, pharmacokinetic/pharmacodynamic (PK/PD) targets have evolved over time with further assessments of the impact of optimized antimicrobial exposures on outcomes. For instance, early *in vitro* time-kill studies identified that maintenance of unbound drug (i.e., free fraction) concentration in serum above the minimum inhibitory concentration (MIC) for 60–70% of the dosing interval (denoted as 60–70% *f*T > MIC) optimized the bactericidal activity of cephalosporins (7). However, additional *in vitro* models have demonstrated the ability to further enhance the bactericidal killing of cephalosporins by both extending the fraction of time above the MIC (e.g., 100% *f*T > MIC), as well as increasing serum concentrations up to 4 times the MIC throughout that period of time (7, 8). Subsequent studies in critically ill adult patients have consistently demonstrated improvements in bacteriologic eradication and clinical cure when antibiotic exposures are increased beyond the more conservative, earlier identified *in vitro* targets (9–13). Further, multiple studies in adult and pediatric populations have shown that traditional, weight-based dosing regimens do not reliably achieve antibiotic targets associated with improved clinical outcomes (10, 14, 15).

Initially developed as a therapy to aid in the removal of fluid and solutes in patients with chronic kidney disease, dialysis now involves numerous modalities of RRT, including intermittent and continuous. While studies have largely failed to demonstrate a difference in outcomes including vasopressor use and mortality between different RRT modalities in critically ill patients (16, 17), the Kidney Disease: Improving Global Outcomes (KDIGO) guidelines recommend CRRT for hemodynamically unstable patients due to its more hemodynamically neutral nature compared to intermittent RRT (18). Under the umbrella of CRRT, several different techniques may be used including continuous veno-venous hemofiltration (CVVH), continuous veno-venous hemodialysis (CVVHD), and continuous veno-venous hemodiafiltration (CVVHDF). Convective clearance is the main driver of solute removal in hemofiltration. Because of this, drug clearance is largely dependent on the ultrafiltration rate (19). Meanwhile, diffusion drives solute removal in hemodialysis, making molecular weight and concentration gradients key parameters that affect drug clearance (20). Both processes occur in hemodiafiltration, thus theoretically generating the highest rate of drug clearance. Despite their differences, each RRT modality is known to affect the clearance of many drugs, particularly those that are hydrophilic with a low volume of distribution (Vd) and have low protein binding (20, 21). Modern high-flux hemofilters have pore sizes exceeding 30 kDa which reduces the effect of molecular weight on RRT clearance for most small molecules, however, molecular weight is an important factor for large molecules and biologics (21).

Many factors are known to influence drug clearance including age, renal function, hepatic function, and use of extracorporeal therapies like RRT (20, 22). Despite mounting evidence for optimal antimicrobial exposures in critically ill patients, with the exception of vancomycin, most commonly used antimicrobials do not routinely undergo routine therapeutic drug monitoring (TDM). The achievement of goal exposures in CRRT is further complicated by the relatively sparse data describing the impact of CRRT on antimicrobials in children. To date there is only a single systematic review of the impact of RRT on the pharmacokinetic parameters of commonly used medications in pediatric CRRT (23). Even with this review in the published literature, a gap exists between the dosing recommendations most readily available in tertiary dosing references used by clinicians to make dosing decisions at the bedside and the most contemporary data that exist in the literature. The goal of this study is to quantify the most commonly used antimicrobial drugs in pediatric patients on CRRT, and to identify gaps between the contemporary literature-based dosing recommendations and those presented in a frequently used dosing reference. We aim to provide an overview of the current state of antimicrobial dosing recommendations in children so that future studies may be best directed to maximize impact on patient care.

## Materials and Methods

### Continuous Renal Replacement Therapy Cases and Antimicrobial Use

To determine the antimicrobials most often used in pediatric CRRT, we utilized data from the Pediatric Health Information System (PHIS) database to identify children who received CRRT and antimicrobials concurrently. The PHIS database is maintained by the Children’s Hospital Association (CHA; Lenexa, KS) and comprises administrative discharge data from 50 freestanding children’s hospitals representing most major U.S. metropolitan areas. Data quality are assured through a joint effort between the PHIS data quality assurance team and participating hospitals. Data are de-identified prior to extraction and analysis (24). The institutional review board at The Children’s Hospital of Philadelphia deemed this study of de-identified data to not constitute human subjects research.

We identified all children (<18 years of age) treated with systemic antimicrobials during CRRT at a PHIS hospital between July 1, 2018 through June 30, 2021. Patients were determined to have received CRRT if they were billed for continuous arteriovenous hemofiltration (CAVH), the PHIS billing designation for CRRT, on at least two consecutive hospital days. Two consecutive billing days of CAVH resource utilization were required to ensure that an individual received CRRT and minimize misclassification. Subjects had to receive at least one systematically administered (enteral or intravenous) antimicrobial on the same day as CRRT to be included.

We then determined the most commonly used antimicrobials by determining the proportion of cases treated with each agent. Drugs of interest were selected if at least 10% of the cases had received the antimicrobial during CRRT; we also chose to include some medications deemed clinically relevant by the authors (e.g., remdesivir). This list of antimicrobials was then utilized for literature searches to evaluate dosing guidance published in the primary literature.

### Tertiary Reference Dosing Guidance

Dosing guidance for each drug of interest during CRRT was assessed using Lexicomp’s Pediatric and Neonatal Lexi-Drug online database (Lexi-Comp Online; Lexi-Comp, Inc., Hudson, OH, United States). We examined (a) whether dosing recommendations for pediatric CRRT were present in this reference, and (b) what data were used to support the recommendations. Lexi-Comp Online was chosen for comparison as it was rated highest by hospital pharmacists among similar tertiary dosing references based on quality of information, performance (when used to answer clinically relevant drug information questions), and usability (25). Compared to other available resources, it was ranked as the preferred database by participants in the study (25).

### Current Evidence

To determine evidence for dosing recommendations in CRRT, literature searches were performed using PubMed (MEDLINE), OVID EMBASE, Cochrane Library, and Web of Science. The search strategy was restricted to pediatric patients from birth to 18 years and developed using keywords: *pharmacokinetics, drug dosing, pediatrics, antimicrobials, and renal replacement therapy*. We also limited use of publications from years 1990 to 2022 to only include results from widely used veno-venous CRRT circuits. The full search strategy can be found in the (Supplementary Appendix).

Studies were included if they met the following pre-defined criteria: (1) study population of ages 18 years and below, including neonates (0–28 days), infants (29 days–1 year of age), children (>1–12 years), and adolescents (>12–18 years), (2) subjects received CRRT such as: CVVH, CVVHD, and CVVHDF. No restrictions on the use of extracorporeal membrane oxygenation (ECMO) were included within this analysis, however, the number of patients receiving ECMO was recorded due to the potential for alteration in drug pharmacokinetics. All study designs, including clinical trials, observational studies, case reports, and case series, were eligible for inclusion. Studies that were excluded were: (1) non-English publications, (2) animal studies, (3) studies including predominantly adult populations with no clear separation of pediatric patients/subjects, and (4) studies of drug removal in patients undergoing CRRT due to acute ingestions. To maximize identification of pertinent manuscripts, the references of included publications were also reviewed, and all potentially relevant articles not identified through the above search strategy were also retrieved and reviewed for inclusion.

### Data Collection

From each identified article, we recorded all relevant CRRT circuit components studied: CRRT machine, circuit filter/tubing, including membrane porosity, flow rates (i.e., blood, dialysate, ultrafiltrate) and replacement fluid flow rate (i.e., pre-filter vs. post-filter replacement). Additionally, the sieving coefficient (SC) or dialysate saturation constant (S_*D*_) was also extracted. This may simply be defined as the ratio between the drug concentration in the effluent fluid to plasma for a CVVH and CVVHD circuit, respectively.

From each included PK study, evidence-based recommendations for antimicrobial dosing during CRRT were extracted by two reviewers (SD and GS). When multiple studies were performed on the same drug of interest, all recommended dosing regimens were recorded to highlight variability across the various studies. Data from studies that assessed drug PK but did not explicitly provide dosing recommendations were not included.

Dosing recommendations extracted from primary literature and the Lexi-Comp Online database were then compared and level of agreement assessed. Agreement between recommendations was defined as: (a) full agreement, when the dose and dosing frequency matched, (b) partial agreement, when there were multiple recommendations in the literature with at least one matching that contained in Lexicomp, and (c) no agreement, when dose and/or dosing frequency differed.

## Results

**FIGURE 1:**
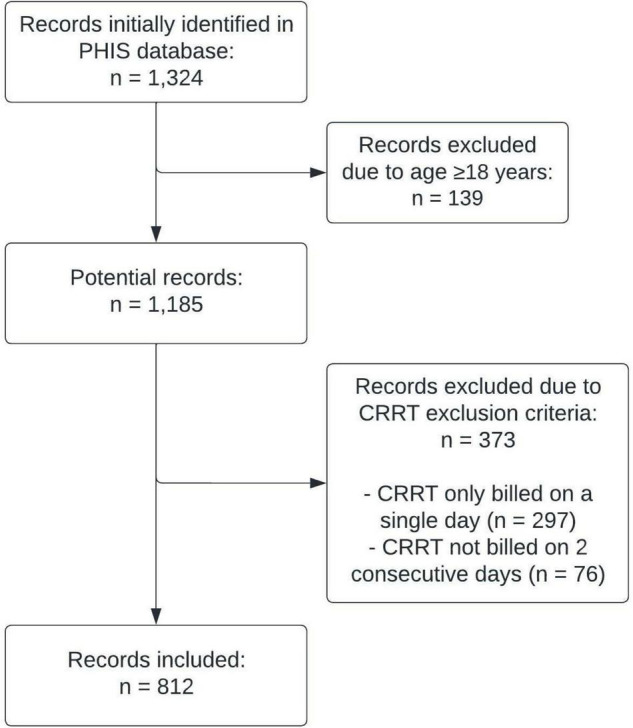
Case ascertainment.

A total of 1,324 individual admissions were identified in the PHIS database during which the patient had ≥1 day of CRRT and antimicrobials were administered based on billing codes. From these initial cases, 139 admissions were excluded due to a patient age of 18 years or older at time of admission, 297 were excluded due to CRRT only being billed on a single day, and 76 were excluded due to CRRT not being billed on 2 consecutive days. The final study sample included 812 individual hospital admissions (Figure 1).

Study patients received care at one of 20 different PHIS hospitals and had a median age of 5 years (range 0–17). The median hospital length of stay was 41 days (interquartile range 20–81). The maximum number of days on which a subject received CRRT and antibiotics was 195 (Table 1).

**TABLE 1:** Patient demographics.

*N*	812
Age, years (median, range)	5 (0–17)
Hospital length of stay, days (median, IQR)	41 (20–81)
Days of concurrent CRRT and DOI (range)	2–195
Number of PHIS hospitals included	20

*CRRT, continuous renal replacement therapy; DOI, drug of interest; PHIS, Pediatric Health Information System.*

A total of 77 unique antimicrobial agents were identified from the 812 hospitalizations and 15 met the threshold of being administered in at least 10% of the cases. Four additional agents were included as drugs with growing clinical relevance: remdesivir, ceftazidime-avibactam, ceftolozane-tazobactam, and daptomycin. Of the included antimicrobial agents, 13 were antibiotics, 3 were antifungals, and 3 were antivirals (Supplementary Table 2). Of the final 19 antimicrobials included, only one (vancomycin) undergoes routine TDM in clinical practice.

Seventeen agents (89.5%) had some type of pediatric CRRT-specific dosing guidance in the Lexi-Comp Online database. Ceftazidime-avibactam lacks pediatric CRRT-specific guidance, though the entry for ceftazidime alone does contain a dosing recommendation. No pediatric CRRT-specific dosing guidance of any kind is present for ceftolozane-tazobactam. Of the 17 agents with pediatric CRRT-specific dosing guidance in Lexi-Comp Online, only a single drug, cefepime, directly references a pediatric study for the included CRRT dosing recommendation–in this case, a non-compartmental analysis of four critically ill children receiving CRRT was used to derive dosing guidance (6).

The most frequently studied drug in children receiving CRRT was meropenem, accounting for nine of the included studies (4, 5, 26–32). Vancomycin followed with three included studies (33–35). The complete details of all included primary literature can be found in Supplementary Table 3. Notably, 5 of the top 10 (50%) most frequently administered antimicrobials in our study have no pediatric-specific CRRT dosing recommendations in the literature (metronidazole, cefazolin, micafungin, trimethoprim-sulfamethoxazole, and ceftriaxone).

The search of primary literature identified 18 studies with dosing recommendations for 8 (42.1%) of the 19 included antimicrobial agents (4–6, 26–41). Of these 8 agents, 2 (cefepime and clindamycin) have complete agreement between dosing recommendations in Lexi-Comp Online and the primary literature. Three agents have no agreement between Lexi-Comp Online and the primary literature (fluconazole, piperacillin-tazobactam, and ceftolozane-tazobactam), and the remaining 3 (vancomycin, meropenem, and linezolid) each have multiple dosing regimens suggested in the literature with at least one of them matching the Lexi-Comp online recommendation (i.e., partial agreement). Of the 18 studies that included dosing recommendations, 8 (44.4%) included 5 patients or less.

## Discussion

As the care of critically ill children advances, the use of extracorporeal therapies is likely to play an increasingly important role. Additionally, as the impact of optimizing antimicrobial agent exposures on patient outcomes continues to gain attention, specific dosing regimens that improve how these drugs are dosed in the critically ill population will be as important as ever. This study highlights both the overall paucity of data for the most commonly used antimicrobial agents in pediatric CRRT, as well as the challenge facing clinicians at the bedside when making dosing decisions for this class of medications.

There are numerous issues to consider when making dosing recommendations for pediatric CRRT. From a practical perspective, only a minority of the drugs used in pediatric CRRT have dosing recommendations based on pediatric CRRT-specific studies. Although adult CRRT literature exists for many drugs, extrapolation of dosing recommendations from adults to children is challenging, especially in such a vulnerable population as one requiring CRRT. This type of extrapolation is routinely performed during the drug development process, incorporating techniques such as allometric scaling and physiologically based PK modeling to account for known differences between populations (42), but these are typically studies involving healthy subjects. Importantly, there are additional considerations in play when doing so among individuals requiring CRRT. For instance, the indications for CRRT among adults and children may differ, leading to importance physiologic differences that can impact PK. When done thoughtfully, extrapolation of dosing recommendations from adults to children is a reasonable first step, providing some degree of dosing guidance to clinicians caring for this special population. However, dedicated PK studies involving children receiving CRRT are needed to develop more informed dosing recommendations.

While the majority of studies assessing PK changes due to CRRT have been performed in adults, some have focused on pediatric PK. Rapp et al. developed a population PK (popPK) model of meropenem in children with a range of renal functions, including those receiving CRRT, and found the use of CRRT to be a significant covariate on meropenem clearance (CL) (28). An additional popPK study assessing meropenem in critically ill children found a 66% increase in Vd for those receiving CRRT (30). These changes in CL and Vd have the potential to significantly impact the drug concentrations achieved and attainment of optimal systemic exposures, a factor shown to be associated with mortality in adults receiving CRRT (43). It is important to consider that physiologic changes that are present in patients requiring antibiotics during CRRT (e.g., those with septic shock) may impact drug PK during CRRT in different ways in adults and children. Thus, clinicians should exercise caution when relying on antimicrobials for which pediatric-specific data are unavailable.

The modality of CRRT used is another important factor to consider when making a dosing recommendation. Many adult studies underpinning published CRRT dose recommendations are dated, using low flux filters and CVVH or CVVHD modalities which may affect drug clearance (44, 45). While modern high-flux hemofilters have likely reduced the effect of specific CRRT modality on drug clearance, significant clearance differences between modalities still exist. This has been demonstrated in adult patients receiving piperacillin-tazobactam and either CVVH or CVVHDF (46). The use of CVVHDF resulted in significantly higher piperacillin clearance compared to CVVH (5.06 ± 1.68 L/h vs. 3.89 ± 1.23 L/h, *p* < 0.05) (46). An additional study compared piperacillin-tazobactam clearance in adult patients receiving CVVH versus CVVHDF (44). While the difference did not reach statistical significance in this study, it may be clinically important as total piperacillin clearance in the CVVHDF group was 7.5 (5.9–11.2) L/h, compared to 4.7 (4.5–9.6) L/h in the CVVH group (*p* = 0.21) (44). Further, mean steady state concentrations in the CVVHDF group were 68.4 (±25.8) mg/L and in the CVVH group 89.1 (±35.6) mg/L (*p* = 0.16) (44).

Further complicating drug dosing pediatric CRRT is that children receiving CRRT span a wide spectrum of body sizes, with patient weights ranging from less 10 kg on the low end and matching that of obese adults on the high end. Although allometric scaling can help account for differences in body sizes when determining drug dosing in healthy patients, body size also influences blood pump speeds, total effluent rates, and pre- vs. post-filter replacement fluids, all of which affect drug clearance (47). Additionally, most pediatric patients receive CRRT using a limited selection of standard adult filters, meaning that many small patients are being dialyzed with relatively large filters. These large filters also have specific prime volumes which may disproportionately affect volume of distribution in smaller patients. And, none of this considers the effects of newer pediatric dialysis technologies, like the *Carpediem*™ or Aquadex FlexFlow^®^ systems, on drug PK.

Our study highlights the challenges of optimal antimicrobial dosing in pediatric CRRT, but solutions are within reach. Therapeutic drug monitoring, the process of measuring drug concentrations to inform dosing, is part of the standard of care for several drugs. However, among the most commonly used drugs in pediatric CRRT that we identified, only vancomycin is routinely subject to TDM in hospitalized children. Expansion of TDM to other commonly used drugs in patients requiring CRRT (e.g., beta-lactam antibiotics) can help confirm appropriate dosing and inform dose adjustments when drug concentrations fall outside of the established target range. Economou et al. demonstrated in an adult population that 35% of CRRT patients receiving beta-lactam antibiotics required dose modification based on TDM in order to achieve target drug exposure (48). We identified several antimicrobials used in pediatric CRRT that have little or no published PK data but that are likely affected by CRRT (e.g., cefepime, piperacillin-tazobactam, fluconazole, cefazolin). Robust study designs able to account for the critical covariates that introduce PK variability, such as age, weight, CRRT effluent rate, underlying disease, and disease severity, are key to formulating empiric pediatric-specific dosing regimens that will optimize drug exposure for patients receiving CRRT. Single-center studies may not be feasible based on CRRT utilization at specific centers, so multicenter studies should be encouraged. Robust trial networks exist that should be leveraged to accelerate the gathering of these data, focusing on the most commonly used antimicrobials first. The Pediatric Trials Network (PTN) has made great strides in closing the gap between available adult and pediatric dosing recommendations. Additionally, the Collaborative Pediatric Critical Care Research Network (CPCCRN) has a strong trial infrastructure from which pediatric PK studies may benefit. It is here that novel study designs centered on opportunistic sampling, microsampling, and popPK techniques hold the potential to improve patient outcomes.

Performing studies that generate data to determine optimal dosing regimens in the pediatric CRRT population is only the first step to address the challenges of optimizing drug dosing for as many children receiving CRRT as possible. The second step, just as important as the first, is ensuring this information is incorporated into tertiary dosing references in a timely manner. It is also critical to define and maintain standards for inclusion in these dosing references. This will ensure the quality of any published recommendations, knowing the impact these recommendations may have on both the safe and efficacious use of medications in a vulnerable population.

Our study has several limitations that are important to put note. The first is that we used the PHIS database. While this database allowed the inclusion of a larger patient population than could be identified at a single institution, which provided us an opportunity to broadly investigate antimicrobial use in pediatric CRRT, it is an administrative database. As such, it lacks sufficient clinical information to examine clinical details about patients who received CRRT or assess the dosing regimens that were utilized. Thus, we could only define antimicrobial use at the agent level and do not know whether doses administered aligned with dosing recommendations provided through the primary literature or Lexi-Comp Online. Second, numerous studies identified through our literature review process had mixed populations, with some receiving CRRT and others not, as well as some patients receiving extracorporeal membrane oxygenation (ECMO) support, which is known to affect drug PK. Thus, dosing guidance provided by these studies did not separate the influence of CRRT from ECMO. Additionally, nearly half of the studies that provided dosing recommendations included 5 or less patients, reducing their power to detect important clinical covariates that may explain interpatient dose-exposure variability. Despite these limitations, our study is the first to assess and quantify the gap in pediatric CRRT dose recommendations between a highly a used tertiary dosing reference, Lexi-Comp Online, and the published literature.

## Conclusion

Significant gaps exist in the data supporting antimicrobial dosing recommendations for children receiving CRRT. Few antimicrobials have robust dosing information available based on pediatric-specific studies. And of those that do, not all are included in bedside references commonly used to inform dosing in clinical practice, such as Lexi-Comp Online. Expanded use of antimicrobial TDM, derivation of population PK models to improve empiric dosing, and leveraging existing clinical trial networks to perform these studies should all be considered to close this information gap. It is vital that future studies be performed that focus on antimicrobial dosing in pediatric CRRT, and that recommendations generated from such studies are promptly incorporated into tertiary dosing references.

## Data Availability Statement

The raw data supporting the conclusions of this article will be made available by the authors, without undue reservation.

## Author Contributions

GS, SD, AE, AZ, KW, and KD: study conception and design. GS, SD, and Y-SH: data collection. GS, SD, Y-SH, and KD: analysis and interpretation of results. All authors contributed to draft manuscript preparation, reviewed the results, and approved the final version of the manuscript.

## Author Disclaimer

The contents are solely the responsibility of the authors and do not necessarily represent the official views of the NICHD or NIH.

## Conflict of Interest

The authors declare that the research was conducted in the absence of any commercial or financial relationships that could be construed as a potential conflict of interest.

## Publisher’s Note

All claims expressed in this article are solely those of the authors and do not necessarily represent those of their affiliated organizations, or those of the publisher, the editors and the reviewers. Any product that may be evaluated in this article, or claim that may be made by its manufacturer, is not guaranteed or endorsed by the publisher.
