# Supplementary material for: Antimicrobial Dosing Recommendations in Pediatric Continuous Renal Replacement Therapy: A Critical Appraisal of Current Evidence
Source: Front Pediatr. 2022 May 12;10:889958. doi: 10.3389/fped.2022.889958 (PMC9134108; doi:10.3389/fped.2022.889958)
Supplement: Supplementary file 1 [file Data_Sheet_1.PDF]

Supplementary Material

1 Supplementary Tables

1.1 Table 2: Summary of Dosing Recommendations for Common Antimicrobials in Pediatric CRRT Ranked by Usage

Drug	Patients Exposed to DOI, n (%)	Lexi-Comp Online Pediatric-Specific CRRT Dose Recommendation?	Primary Pediatric Literature Cited in Lexicomp?	Lexi-Comp Online Recommendation	Primary Literature Recommendation	Recommendation Agreement
Vancomycin	458 (56.4)	Y	N	Infants, Children, and Adolescents:<br>• 10 mg/kg/dose every 12 to 24 hours; monitor serum concentrations	Cies et al. (2016):<br>• 15-20 mg/kg IV loading dose, followed by addition into CRRT solution at a final concentration of 18-30 mg/L<br>Moffet et al. (2019):<br>• 40-50 mg/kg/day Q8-12H based on fat-free mass<br>Zylbersztajn et al. (2018):<br>• RRT without AKI: 40 mg/kg/day Q6H<br>• ECMO with AKI and with or without RRT: 20 mg/kg/day Q12H	Partial
Cefepime	450 (55.4)	Y	Y	Infants, Children, Adolescents:<br>• 50 mg/kg/dose (maximum dose: 2,000 mg/dose) every 8 to 12 hours	Stitt et al. (2019)<br>• Doses > 50 mg/kg Q12H may be required	Full
Metronidazole	233 (28.7)	Y	N	Infants, Children, and Adolescents:<br>• Continuous renal replacement therapy (CRRT): No adjustment required	No recommendation	N/A
Meropenem	231 (28.4)	Y	N	Infants, Children, and Adolescents:<br>• 20 to 40 mg/kg/dose every 12 hours	Cies et al. (2016):<br>• 40 mg/kg bolus, followed by 10 mg/kg/hr CI<br>Nehus (2014):<br>• >5y: 20 mg/kg Q12H, <5y: 20 mg/kg Q8H<br>Nehus et al. (2016):<br>• 20 mg/kg Q12H<br>Rapp et al. (2019):<br>• 50% fT>MIC (<2 μg/mL): 60 mg/kg/day Q8H<br>• 50% fT>MIC (>4 μg/mL): 60 mg/kg/day CI	Partial

					Saito et al. (2020):<br>• 200-300 mg/kg/day Q8H as 1-hr infusion OR 200-300 mg/kg/day Q8H as 3-hr infusion<br><br>Tan et al. (2021):<br>• 20 mg/kg Q8H over 4-hr infusion OR 40 mg/kg Q8H over 2-hr infusion<br><br>Wang et al. (2021):<br>• 20 mg/kg Q8H as a 1-hr infusion<br><br>Wang et al. (2021):<br>• 50-100% T>MIC: 20 mg/kg Q8H IV as 1-h infusion based on BW, eCRCL and MIC; MIC >4 μg/mL: 40 mg/kg Q8H IV as 3-h infusion<br><br>Saito et al. (2021):<br>• 20-40 mg/kg/dose Q8-12H depending on residual renal function (see Appendix 2)	
Fluconazole	204 (25.1)	Y	N	Children and Adolescents:<br>• <1,500 mL/m2/hour (<25 mL/m2/minute):<br>o Loading dose: Usual dose: 6 to 10 mg/kg/dose once<br>o Maintenance dose: 3 to 12 mg/kg/dose once daily depending on indication<br>• ≥1,500 mL/m2/hour (≥25 mL/m2/minute):<br>o Loading dose: Usual dose: 6 to 10 mg/kg/dose once<br><br>Maintenance dose: 6 to 12 mg/kg/dose once daily depending on indication	Oualha et al. (2019):<br>• 900 mg loading dose, followed by 600 mg IV BID	No
Cefazolin	203 (25)	Y	N	Infants, Children, and Adolescents:<br>• 25 to 50 mg/kg/dose IV every 8 to 12 hours, maximum dose: 2,000 mg/dose	No recommendation	N/A
Piperacillin-tazobactam	182 (22.4)	Y	N	Infants, Children, and Adolescents:<br><br>35 to 50 mg piperacillin/kg/dose every 8 hours	Tang Girdwood et al. (2020):<br>• 80 mg/kg Q6H	No
Micafungin	172 (21.2)	Y	N	No dosage adjustment necessary	No recommendation	N/A

Trimethoprim-sulfamethoxazole	145 (17.9)	Y	N	General Dosing, Children and Adolescents:<br>• Combined dialysis flow + ultrafiltration rate <1,500 mL/m2/hour: 3 to 5 mg TMP/kg/dose every 18 hours<br>• Combined dialysis flow + ultrafiltration rate ≥ 1,500 mL/m2/hour: 4 to 5 mg TMP/kg/dose every 18 hours<br><br>Pneumocystis jirovecii pneumonia (PCP): Treatment, Children:<br>• 5 mg TMP/kg/dose every 8 hours	No recommendation	N/A
Ceftriaxone	113 (13.9)	Y	N	Not dialyzable	No recommendation	N/A
Clindamycin	105 (12.9)	Y	N	Infants, Children, and Adolescents:<br>• No dosage adjustment necessary	Poli et al. (2019):<br>• No dose adjustment necessary	Full
Ganciclovir	103 (12.7)	Y	N	Infants, Children, and Adolescents:<br>• Based on experience in adult patients, dosage adjustment necessary.	No recommendation	N/A
Amphotericin B	88 (10.8)	Y	N	AmBisome:<br>• Poorly dialyzed<br>Abelcet - Infants, Children, and Adolescents:<br>• No supplemental dosage necessary<br>Amphotericin deoxycholate - Infants, Children, and Adolescents:<br>Renal replacement therapy: Poorly dialyzed; no dosage adjustment necessary	No recommendation	N/A
Acyclovir	84 (10.3)	Y	N	Infants, Children and Adolescents:<br>• 10 mg/kg/dose every 12 hours	No recommendation	N/A
Linezolid	82 (10.1)	Y	N	Infants, Children, and Adolescents:<br>• No adjustment necessary	Yang et al. (2021):<br>• <12 y: 10 mg/kg IV Q8H with MIC ≤ 1 µg/mL<br>• 12-18 y: 600 mg IV Q12H with MIC ≤ 1 µg/mL<br>• <12y with AST >200 U/L: 10 mg/kg IV Q12H with MIC ≤ 1 µg/mL<br>• 12-18y with AST >200 U/L: 600 IV Q48H with MIC ≤ 1 µg/mL	Partial
Ceftazidime-avibactam	9 (1.1)	N	N	No recommendation	No recommendation	N/A
Daptomycin	9 (1.1)	Y	N	Infants, Children, and Adolescents:<br>• 8 mg/kg/dose every 48 hours; monitor CPK weekly	No recommendation	N/A

Remdesivir	6 (0.7)	Y	N	Infants, Children, and Adolescents weighing ≥3.5 kg (FDA 2020a; manufacturer's labeling):<br><br>eGFR <30 mL/minute: No formal safety or pharmacokinetic data are available for patients with kidney impairment or who are receiving renal replacement therapies (Barlow 2020; manufacturer's labeling). Use is not recommended by the manufacturer. However, significant toxicity with a short duration of therapy (eg, 5 to 10 days) is unlikely; benefits may outweigh the risks in select patients (Adamsick 2020). In retrospective studies in adult patients with acute or chronic kidney impairment, remdesivir administered at the recommended dose has not been shown to routinely increase serum creatinine or cause severe hepatic toxicity (ALT >5 times the normal limit) (Ackley 2020; Estiverne 2020; Pettit 2020; Thakare 2020).	No recommendation	N/A
Ceftolozane-tazobactam	4 (0.5)	N	N	No recommendation	Collignon et al. (2021):<br>• 150 mg/kg/day IV administered as CI for treatment of *Pseudomonas aeruginosa*<br>Butragueño-Laiseca et al. (2020):<br>• 30 mg/kg IV Q8H	No

1.2 Table 3: Summary of Evidence

**Author**<br>(Year)	**Antimicrobial**	**Dose**	**Study Design**	***N***	**Demographics**<br>[range]	**Type of illness**	**CRRT,**<br>Received CRRT/ no. participants	**Flow Rates**	**ECMO,**<br>Received ECMO/ no. participants
Penicillins									
Tang Girdwood et al. (2020)[37]	Piperacillin-tazobactam	48 mg/kg IV Q8H over 30 minutes	Case Report	1	Age:<br>13 y	Sepsis	CVVHD: 1/1	Blood:<br>150 mL/min	No

					Weight:<br>42 kg		in addition to MARS	Pre-filter:<br>150 mL/h	
Cephalosporins									
Stitt et al.<br>(2019)[6]	Cefepime	48-64 mg/kg IV Q6-12H	Retrospective	4	Age:<br>1.8 y [0.5-5]<br>Weight:<br>11.6 kg<br>[5.4-25]	Mixed	CVVHDF: 4/4<br>HF 1000 filter	Blood:<br>75 mL/min<br>Pre-filter: 512.5 mL/h<br>Post-filter:<br>50 mL/h<br>Dialysate:<br>925 mL/h<br>Effluent:<br>4986 mL/1.73m^2/h	No
Collignon et al. (2021)[40]	Ceftolozane-Tazobactam	150 mg/kg/day IV administered as CI	Case Report	1	Age:<br>15 m	Pneumonia/sepsis	CVVHDF: 1/1<br>Prismaflex	Blood:<br>4.1-6.9 mL/kg/min	No

					Weight:<br><br>NR			Replacement:<br><br>41.6-69.4 mL/kg/h<br><br>Net ultrafiltration:<br><br>3.4-9.7 mL/kg/h	
Butragueño-Laiseca et al.<br><br>(2020)[41]	Ceftolozane-Tazobactam	No AKI:<br><br>40 mg/kg IV Q6H<br><br>AKI:<br><br>36 mg/kg IV Q8H<br><br>CVVHDF:<br><br>30 mg/kg IV Q8H	Population PK	3	Age:<br><br>12 m [8-19]<br><br>Weight:<br><br>8.5 kg [5.8-11]	Pneumonia	CVVHDF: 1/3<br><br>Prismaflex	Blood:<br><br>30 mL/min<br><br>Replacement:<br><br>160 mL/h<br><br>Dialysate:<br><br>250 mL/h<br><br>Effluent:<br><br>470 mL/h	No
**Carbapenems**									
Cies et al.<br><br>(2016)[26]	Meropenem	40 mg/kg IV bolus, followed by 10 mg/kg/hr CI	Case Report	1	Age:<br><br>10 d	Septic shock	CVVHDF: 1/1<br><br>AN 69 filter	Blood:<br><br>50 mL/min	ECMO: 1/1

					Weight:<br><br>2.8 kg			Pre-filter:<br><br>250 mL/h<br><br>Post-Filter:<br><br>50 mL/hr<br><br>Dialysate:<br><br>200 mL/hr<br><br>Ultrafiltration:<br>0-40 mL/hr	
Nehus et al.<br><br>(2014)[27]	Meropenem	20 mg/kg IV Q8-12H	Population PK	287	Age:<br><br><1y: 52<br><br>1-5y: 58<br><br>5-12: 69<br><br>12-18y: 79<br><br>>18y: 29<br><br>Weight:<br><br>Median:<br><br><1y: 3.7<br><br>(IQR 2.9-5.8)	Mixed	CVVHD: 140/287<br><br>CVVH:<br><br>78/287<br><br>CVVHDF: 69/287<br><br>M100 0.9m^2 and M60 0.6m^2 polyacrylonitrile	Blood:<br><br><1y:<br><br>40 mL/min<br><br>1-5y:<br><br>62.5 mL/min<br><br>5-12y:<br><br>100 mL/min<br><br>12-18y:	No

					1-5y :14<br><br>(IQR 11.3-16.5)<br><br>5-12y: 31.6<br><br>(IQR 22.0-37.8)<br><br>12-18y: 59.7<br><br>(IQR 44.6-70.4)<br><br>>18: 55<br><br>(IQR 50.0-78.5)			150 mL/min<br><br>>18y:<br><br>120 mL/min<br><br>Ultrafiltration:<br><br><1y:<br><br>8.5 mL/kg/h<br><br>1-5y:<br><br>7.4 mL/kg/h<br><br>5-12:<br><br>4.7 mL/kg/h<br><br>12-18:<br><br>4.1 mL/kg/h<br><br>>18:<br><br>2.8 mL/kg/h<br><br>Total effluent:	

								<1y:<br>107.6 mL/kg/h<br><br>1-5y:<br>57.4 mL/kg/h<br><br>5-12y:<br>43.3 mL/kg/h<br><br>12-18:<br>35.4 mL/kg/h<br><br>>18y:<br>24.4 mL/kg/h	
Nehus et al.<br>(2016)[4]	Meropenem	Mean: 18.6 mg/kg IV Q12H	Prospective, open-label	7	Age:<br>14.3y<br>[5-21y]<br><br>Weight:<br>18.59 kg	Mixed	CVVH: 2/7<br>CVVHDF: 5/7<br><br>AN 69 (0.6 or $0.9m^2$)<br>HF 1000 ($1.1m^2$)	**CVVH:**<br>Blood:<br>175 mL/min<br><br>Pre-filter:<br>1797 mL/min	ECMO: 1/7

					[21.9-72.6]			Post-filter:<br><br>185 mL/min<br><br>Ultrafiltration:<br>211 mL/min<br><br>**CVVHDF:**<br><br>Blood:<br><br>164 mL/min<br><br>Pre-filter:<br><br>706 mL/min<br><br>Post-filter:<br><br>50 mL/min<br><br>Dialysate:<br><br>760 mL/min<br><br>Ultrafiltration:<br>526.6 mL/min	

Rapp et al. (2020)[28]	Meropenem	60 mg/kg/day IV Q8H or CI	Population PK	40	Age:<br><br>Median:<br>16.8 m [1.4-187.2]<br><br>Weight:<br>Median:<br>9.1 kg<br>[3.8-59]	Mixed	CRRT: 11/40<br><br>Modality NR	NR	ECMO: 8/40<br><br>ECMO+CRRT: 2/40
Saito et al.<br>(2020)[29]	Meropenem	300 mg/kg/day IV Q8H as a 3-h infusion	Case Report	1	Age:<br>19 m<br><br>Weight:<br>9 kg	ESBL bacteremia	CVVHD: 1/1<br>UT filter 300S ($0.3m^2$)	Blood:<br>50-80 mL/min<br><br>Dialysate:<br>820-1500 mL/h	VA-ECMO: 1/1
Tan et al.<br>(2021)[31]	Meropenem	20-40 mg/kg IV Q12H	Prospective, open-label, population PK	9	Age:<br>7.28y<br>[0.1-18.9]<br><br>Weight:<br>24.52	Mixed	CVVH: 4/9<br>CVVHDF: 5/9<br><br><10kg: PAES ($0.2m^2$)	Dialysate: 737.4 $mL/1.73m^2/h$<br><br>Ultrafiltration: 1952 $mL/1.73m^2/h$	VV-ECMO: 3/9<br><br>VA-ECMO: 1/9

					[2.6-56.3]		11-30 kg: AN69 ($0.6m^2$)<br><br>>30kg: AN69 ($0.9m^2$)		
Wang et al.<br><br>(2021)[5]	Meropenem	20 mg/kg IV Q8H as a 1-hr infusion	Prospective, observational	27	Age:<br><br>Median:<br><br>1.29 y<br><br>(IQR 0.67-3.25y)[d]<br><br>2.0 y<br><br>(IQR 1.13-6.68y)[e]<br><br>2.50 y<br><br>(IQR 0.5-5.25y)[f]<br><br>Weight:<br><br>Median:<br><br>11kg<br><br>(IQR 7.75-12.63)[d]	Sepsis	CVVHDF: 6/27<br><br>AN 69 filter	Blood:<br><br>4 mL/kg/min<br><br>Pre-filter:<br><br>25 mL/kg/h<br><br>Dialysate:<br><br>20 mL/kg/h<br><br>Ultrafiltration: 2 mL/kg/h	VA-ECMO: 6/27

					12.50 kg<br><br>(IQR 8.6-27.15)[e]<br><br>14.50 kg<br><br>(IQR 6.53-21.50)[f]				
Jabareen et al. (2021)[49]	Meropenem	40 mg/kg/dose IV Q8H, subsequently switched to 240 mg/kg/d IV as a CI	Case Report	1	PNA:<br><br>29 d<br><br>GA:<br><br>35 w<br><br>Weight:<br><br>2.5 kg	Sepsis	CVVHDF: 1/1<br><br>Prismaflex M60<br><br>AN 69 filter	Blood:<br><br>20-50 mL/min<br><br>Pre-filter:<br><br>70-100 mL/h<br><br>Post-filter:<br><br>20-100 mL/h<br><br>Dialysate:<br><br>20-100 mL/h<br><br>Ultrafiltration: 40-70 mL/h	VA-ECMO: 1/1

Wang et al. (2021)[32]	Meropenem	20-40 mg/kg Q8H IV as a 1-3 h infusion	Prospective, observational Population PK	25	Age:<br>2 y<br>[0.71-3.88]<br><br>Weight:<br>11.50 kg<br>[9.5-36.3]	**Sepsis**	CVVHDF: 13/25<br>Prismaflex<br>AN 69 filter	NR	VA-ECMO: 13/25<br><br>ECMO + CRRT:<br>4/25
Saito et al. (2021)[30]	Meropenem	Median:105.2 mg/kg/day<br>[40-293.4] as a 1-3h infusion	Retrospective, Population PK	34	Age:<br>Median:<br>1.4 y<br>[0.03-14.6]<br><br>Weight:<br>Median:<br>8.9 kg<br>[2.7-40.9]	Mixed	CVVHDF: 6/34<br><br>CVVHD: 2/34	Blood:<br>Median:<br>40 mL/min<br>[15-80]<br><br>Dialysate:<br>Median:<br>1600 mL/h<br>[600-4100]<br><br>Ultrafiltration:<br>Median:<br>1600 mL/h<br>[600-8400]	ECMO + CVVHDF: 3/34

**Glycopeptides**									
Armstrong et al. (1993)[50]	Vancomycin	11-15 mg/kg/dose IV	Case Report	1	Age:<br>4 m<br>Weight:<br>4 kg	Sepsis	CVVH: 1/1<br>Amicon dialfilter	Blood:<br>15 mL/min<br>Ultrafiltration: 20-30 mL/min	No
Cies et al. (2016)[33]	Vancomycin	15-20 mg/kg IV loading dose, followed by addition into CRRT solution at a final concentration of 18-30 mg/L	Case-series	11	Age:<br>8.38y<br>[0.08-18y]<br>Weight:<br>35.72<br>[3.1-61.7]	Mixed	CVVH: 7/11*<br>CVVHD: 2/11*<br>CVVHDF: 5/11*<br>AN69 filter<br>*patients received multiple methods of CRRT	**CVVH:**<br>Blood:<br>215.39 mL/min<br>Pre-filter: 773.08 mL/h<br>Post-filter: 159.01 mL/h<br>**CVVHD:**<br>Blood:<br>440 mL/min<br>Pre-filter:<br>100 mL/h<br>Post-filter:	No

								50 mL/h<br><br>Dialysate:<br><br>1100 mL/h<br><br>**CVVHDF:**<br><br>Blood:<br><br>218 mL/min<br><br>Pre-filter: 977.5 mL/h<br><br>Post-filter: 223.68 mL/h<br><br>Dialysate:<br><br>800 mL/h	
Moffet et al.<br><br>(2019)[34]	Vancomycin	Mean dose: 14.3 mg/kg/dose IV	Retrospective, population PK	138	Age:<br><br>Median:<br><br>4.9 y<br><br>(IQR 1.0-14.5y)<br><br>GA: 40 w<br><br>(IQR 39-40w)	NR	CVVHDF: 138/138<br><br>HF 1000 filter	Blood:<br><br>102 mL/min<br><br>Pre-filter:<br><br>464 mL/h<br><br>Post-filter:<br><br>48 mL/h	No

					PMA: 297w<br><br>(IQR 93-791w)<br><br>Weight:<br><br>31.0 ±25.8			Dialysate:<br><br>598 mL/h<br><br>Ultrafiltration: 289 mL/h	
Zylbersztajn et al. (2018)[35]	Vancomycin	ECMO no AKI/RRT: 40 mg/kg/day IV Q6H<br><br>ECMO AKI with or without RRT: 40 mg/kg/d IV Q8H<br><br>ECMO with RRT without AKI: 40 mg/kg/d IV Q8H	Retrospective	29	Age:<br><br>Median:<br><br>24 m<br><br>(IQR 10-100m)[a]<br><br>42 m<br><br>(IQR 12-99m)[b]<br><br>8 m<br><br>(IQR 1-72m)[c]<br><br>Weight:<br><br>Median:<br><br>12<br><br>(IQR 8-21)[a]	Suspected or confirmed infection due to gram positive cocci	CRRT: 11/29<br><br>Modality NR	NR	VV-ECMO: 19/29*<br><br>VA-ECMO: 20/29*<br><br>*Patients may have received both methods of ECMO

					13.5<br><br>(IQR 8-23)[b]<br><br><br>8<br><br>(IQR 2.9-23)[c]				
**Lincosamides**									
Poli et al. (2019)[38]	Clindamycin	NR	Case Report	1	Age:<br><br>14 y<br><br><br>Weight:<br><br>75 kg	Septic shock	CVVH: 1/1<br><br><br>Cytosorb cartridge	NR	VV/VA-ECMO: 1/1
**Oxazolidinones**									
Yang et al.<br><br>(2021)[39]	Linezolid	<12 y:<br><br>10 mg/kg IV Q8H<br><br><br>12-18y:<br><br>600 mg IV Q12H	Prospective, open-label, population PK	63	Age:<br><br>5.21y ±4.22<br><br>[0.10-15.3]<br><br><br>Weight:<br><br>22.28±15<br><br>[4.20-70]	Critically ill patients with staphylococcal infections	CRRT: 15/63	NR	ECMO: 2/63
**Antivirals**									

Cies et al. (2015)[51]	Acyclovir	30 mg/kg/dose IV Q8H, then added to dialysate solution for final concentration of 5.5 ug/mL	Case Report	1	Age:<br>14 d<br>Weight:<br>NR	Disseminated HSV	CRRT: 1/1<br>Modality NR	NR	ECMO: 1/1
**Antifungals**									
Oualha et al. (2019)[36]	Fluconazole	10 mg/kg/day IV	Case Report	1	Age:<br>17 y<br>Weight:<br>NR	Fungal cholecystitis	CVVHD: 1/1	Blood:<br>150 mL/min<br>Dialysate:<br>4000 mL/h<br>Ultrafiltration: 90 mL/h	No

2 Appendix

2.1 Appendix 1: Overview of final search strategy with included databases and syntax

Database	Search Syntax
PubMed	(pharmacokinetics[mesh] OR pharmacokinetic*[tw] OR “drug kinetic*”[tw] OR ADME[tw] OR “biological availability”[tw] OR “area under curve”[tw] OR biotransformation[tw] OR "drug clearance"[tw] OR “metabolic clearance rate”[tw] OR “drug absorption”[tw] OR “tissue distribution”[tw] OR

	"volume of distribution"[tw] OR “drug elimination”[tw] OR “drug liberation”[tw] OR “drug metabolism”[tw] OR "drug dose"[tw] OR “therapeutic equivalency”[tw]) **AND** (infant, newborn[mesh] OR child[mesh] OR pediatrics[mesh] OR neonat*[tw] OR infant*[tw] OR newborn*[tw] OR pediatric*[tw] OR paediatric*[tw] OR child*[tw]) **AND** (renal replacement therapy[mesh] OR hemodialys*[tw] OR “peritoneal dialysis”[tw] OR “extracorporeal dialysis”[tw] OR hemodiafiltration[tw] OR hemofiltration[tw] OR "renal replacement therapy"[tw] OR “kidney replacement therapy”[tw] OR "continuous renal replacement therapy"[tw] OR "continuous veno-venous hemodialysis"[tw] OR "continuous veno-venous hemofiltration"[tw] OR "continuous veno-venous hemodiafiltration”[tw] OR “intensive care”[mesh] OR “critical care”[tw] OR “ICU”[tw] OR “PICU”[tw] OR “NICU”[tw])
EMBASE	1. exp pharmacokinetics/<br>2. exp bioavailability/<br>3. exp area under the curve/<br>4. (pharmacokinetic* or drug kinetic* or ADME or biological availability or area under curve or biotransformation or drug clearance or metabolic clearance rate or drug absorption or tissue distribution or volume of distribution or drug elimination or drug liberation or drug metabolism or drug dose or therapeutic equivalency).tw.<br>5. 1 or 2 or 3 or 4<br>6. exp newborn/<br>7. exp child/<br>8. (neonat* or infant* or newborn* or pediatric* or paediatric* or child*).tw.<br>9. 6 or 7 or 8<br>10. exp renal replacement therapy/<br>11. (hemodialys* or peritoneal dialysis or extracorporeal dialysis or hemodiafiltration or hemofiltration or renal replacement therapy or kidney replacement therapy or continuous renal replacement therapy or

	continuous veno venous hemodialysis or continuous veno venous hemofiltration or continuous veno venous hemodiafiltration).tw.<br>12. 10 or 11<br>13. 5 and 9 and 12<br>14. limit 13 to english language
Cochrane Library	(pharmacokinetic* OR “drug kinetic*” OR ADME OR “biological availability” OR “area under curve” OR biotransformation OR "drug clearance" OR “metabolic clearance rate” OR “drug absorption” OR “tissue distribution” OR "volume of distribution" OR “drug elimination” OR “drug liberation” OR “drug metabolism” OR "drug dose" OR “therapeutic equivalency”):ti,ab,kw (neonat* OR infant* OR newborn* OR pediatric* OR paediatric* OR child*):ti,ab,kw (hemodialys* OR "peritoneal dialysis" OR "extracorporeal dialysis" OR hemodiafiltration OR hemofiltration OR "renal replacement therapy" OR "kidney replacement therapy" OR "continuous renal replacement therapy" OR "continuous veno venous hemodialysis" OR "continuous veno venous hemofiltration" OR "continuous veno venous hemodiafiltration"):ti,ab,kw

2.2 Appendix 2: Probability of Target Attainment at Various *f*T>MIC Targets from Saito J, et al. Antimicrob Agents Chemother. 2021;65(2).

				PTA (%) for SCr-based eGFR (mL/min) (standard dosing regimen)															
				MIC = 1 µg/mL				MIC = 2 µg/mL				MIC = 4 µg/mL				MIC = 8 µg/mL			
Infusion Duration (hours)	*f*T>MIC (%)	SIRS status	CRRT status	<10 (20 mg/kg q24h)	10-25 (20 mg/kg q12h)	26-50 (40 mg/kg q12h)	>50 (40 mg/kg q8h)	<10 (20 mg/kg q24h)	10-25 (20 mg/kg q12h)	26-50 (40 mg/kg q12h)	>50 (40 mg/kg q8h)	<10 (20 mg/kg q24h)	10-25 (20 mg/kg q12h)	26-50 (40 mg/kg q12h)	>50 (40 mg/kg q8h)	<10 (20 mg/kg q24h)	10-25 (20 mg/kg q12h)	26-50 (40 mg/kg q12h)	>50 (40 mg/kg q8h)
0.5	40	-	-	73.2	100	100	100	12.3	100	100	100	0.1	36.5	75	100	0	0.1	9	26.3
		-	+	100	100	100	100	73.7	100	100	100	2.6	93.3	100	100	0	1.3	48.2	100

		+	-	5.8	89.2	100	100	0.1	30.2	51.9	100	0	1.1	5.7	19	0	0	0	0
		+	+	63.7	100	100	100	6.1	100	100	100	0	12.7	66.4	100	0	0	1.9	0.6
	100	-	-	0.1	10.5	21.3	79.8	0	0.4	1.5	24.8	0	0	0	1.7	0	0	0	0
		-	+	1.3	64.9	82.6	100	0	9.9	24.8	89.8	0	0.1	1.2	30.4	0	0	0	0.7
		+	-	0	0.1	0.7	11.6	0	0	0	0.5	0	0	0	0	0	0	0	0
		+	+	0	4.8	9.8	71.4	0	0.1	0.5	17	0	0	0	0.7	0	0	0	0
3	40	-	-	100	100	100	100	30.8	100	100	100	0.6	97.5	100	100	0	1.7	52.5	100
		-	+	100	100	100	100	98.9	100	100	100	6.3	100	100	100	0	2.2	89.4	100
		+	-	39.6	100	100	100	5.4	20.1	100	100	0	16.2	68.4	100	0	0	2.3	100
		+	+	96.5	100	100	100	17.1	100	100	100	0	42	100	100	0	0	10.7	100
	100	-	-	0.1	21	36	100	0	1.1	4.6	59.9	0	0	0.1	9.1	0	0	0	0.1
		-	+	2.5	90.5	100	100	0	23.1	45.7	100	0	0.3	4.5	70.3	0	0	0	5.8
		+	-	0	0.4	1.6	33.2	0	0	0	3.5	0	0	0	0.1	0	0	0	0
		+	+	0	13.2	23.6	89.1	0	0.4	2	52.7	0	0	0	5.3	0	0	0	0

Shaded values indicate a probability of target attainment of >90%. SCr, serum creatinine; eGFR, estimated glomerular filtration rate; SIRS, systemic inflammatory response syndrome; CRRT, continuous renal replacement therapy
